# Recent Progress on Exosomes in RNA Virus Infection

**DOI:** 10.3390/v13020256

**Published:** 2021-02-08

**Authors:** Liying Zhang, Yichen Ju, Si Chen, Linzhu Ren

**Affiliations:** College of Animal Sciences, Key Lab for Zoonoses Research, Ministry of Education, Jilin University, Changchun 130062, China; zhangliy@jlu.edu.cn (L.Z.); gustavo1221@sina.com (Y.J.); chensi_1024@163.com (S.C.)

**Keywords:** exosome, RNA virus, infection

## Abstract

Recent research indicates that most tissue and cell types can secrete and release membrane-enclosed small vesicles, known as exosomes, whose content reflects the physiological/pathological state of the cells from which they originate. These exosomes participate in the communication and cell-to-cell transfer of biologically active proteins, lipids, and nucleic acids. Studies of RNA viruses have demonstrated that exosomes release regulatory factors from infected cells and deliver other functional host genetic elements to neighboring cells, and these functions are involved in the infection process and modulate the cellular responses. This review provides an overview of the biogenesis, composition, and some of the most striking functions of exosome secretion and identifies physiological/pathological areas in need of further research. While initial indications suggest that exosome-mediated pathways operate in vivo, the exosome mechanisms involved in the related effects still need to be clarified. The current review focuses on the role of exosomes in RNA virus infections, with an emphasis on the potential contributions of exosomes to pathogenesis.

## 1. Introduction

Extracellular vesicles (EVs) are small lipid bilayer-enclosed nanoparticles that are released from multiple types of cells [1,2,3]. The content, size, and membrane composition of EVs are highly heterogeneous and dynamic and are affected by the cellular source, state, and environmental conditions [4]. In recent decades, several EVs, including microvesicles, apoptotic bodies, and exosomes, have been found to play important roles in various physiological and pathological processes [3,4].

Exosomes were first identified by Harding et al. in 1983 and confirmed by Johnstone et al. in 1987 [5,6]. As reported, multivesicular bodies (MVBs) are formed by endosome budding in the cytoplasm [3], while exosomes are released into the extracellular fluids by fusion of MVBs with the cell surface, resulting in secretion [7]. The size of exosomes ranges from 30–120 nm, and exosomes contain a variety of proteins, lipids, and nucleic acids. Exosomes are important intercellular communicators because of their roles in transferring signals and materials. Depending on the cell type, many cellular functions have been attributed to exosomes [7]. Importantly, increasing evidence indicates that exosomes play complex roles in viral diseases [8,9]. For example, exosomes can enhance viral infection by spreading viral components, such as proteins and nucleic acids, and inhibit the immune response or promote immune escape [7,10,11,12]. They can also inhibit infection by triggering innate or acquired immunity and eliminating pathogens via induction of apoptosis or other signaling pathways [7,10,11,12]. In addition, the viral replication cycle from virus entry to assembly and release also involves the exosomal pathway [7,10,11]. At present, there are still no effective preventative, therapeutic, or even diagnostic methods for many viral diseases, including human immunodeficiency virus (HIV) infection, viral hepatitis, and carcinoma. Exosomes may be used as markers for disease diagnosis, vehicles for treatment, or targets for treatment or vaccine research. Moreover, further research on the relationships between viruses and exosomes is warranted.

## 2. Exosome Biogenesis

Johnstone first coined the term exosome to describe the process that underlies the transformation from a reticulocyte to a mature erythrocyte. Because the vesicular secretion process was similar to “reverse endocytosis,” small vesicles were termed “exosomes” [6]. Exosomes are formed within the endosomal network, and early endosomes have three fates: recycling, degradation, and exocytosis. The remainder becomes late endosomes, also called MVBs, because of the observation that an MVB contains many small lysosomes to target related proteins and lipids for degradation; the second fate is fusion with the plasma membrane to release internal vesicles into the extracellular milieu, which then are considered exosomes, and the last fate is to remain in the cytoplasm and storage compartments [10]. Although the exact mechanism of how this process is regulated is still unclear, scientists have established many theories of exosome biogenesis, including endosomal sorting complex required for transport (ESCRT)-dependent [13] and ESCRT-independent pathways [14], which include the oligomerization of tetraspanin complexes [15], the sphingomyelinase pathway that catalyzes ceramide synthesis [16], or phospholipase D2 and adenosine diphosphate (ADP) ribosylation factor-6-mediated intraluminal vesicle (ILV) budding [17].

**Figure 1:**
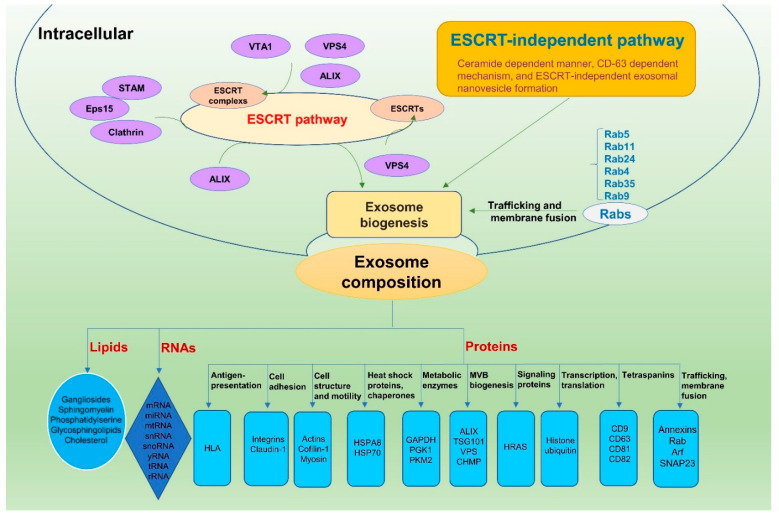
Exosome biogenesis and composition. Exosomes are formed within the endosomal network via ESCRT-dependent and ESCRT-independent pathways and fuse with the plasma membrane for release into the extracellular space. Moreover, Rab GTPases are also involved in the trafficking and membrane fusion steps of exosome formation. Exosomes are rich in a wide variety of proteins, lipids, and nucleic acids, which depend on the type of cell and physiological or pathological state. Abbreviations: ADP-ribosylation factor (Arf), ALG-2 interacting protein X (ALIX), charged multivesicular body protein (CHMP), cluster of differentiation (CD), endosomal sorting complex required for transport (ESCRT), epidermal growth factor receptor pathway substrate 15 (Eps15), glyceraldehyde-3-phosphate dehydrogenase (GAPDH), heat shock protein (HSP), hepatocyte growth factor-regulated tyrosine kinase substrate (HRS), HRas proto-oncogene, GTPase (HRAS), Human leukocyte (HLA), multivesicular body (MVB), phosphoglycerate kinase (PGK), pyruvate kinase M (PKM), Rab GTPases (Rab), signal transducing adaptor molecule (STAM), Synaptosomal-associated protein 23 (SNAP23), tumor susceptibility 101 (TSG101), vacuolar protein sorting 4 (VPS4), Vacuolar protein-sorting-associated proteins (VPS), and vesicle trafficking 1 (VTA1).

ESCRTs consist of approximately twenty proteins that assemble into four complexes (ESCRT-0, ESCRT-I, ESCRT-II, and ESCRT-III) with the associated proteins vacuolar protein sorting 4 (VPS4), vesicle trafficking 1 (VTA1), and ALG-2 interacting protein X (ALIX) (Figure 1). The ESCRT-0 complex recognizes and sequesters ubiquitylated proteins in the endosomal membrane, whereas the ESCRT-I and ESCRT-II complexes appear to be responsible for inward membrane budding with sequestered cargo, and ESCRT-III components subsequently drive vesicle scission. ESCRT-0 comprises hepatocyte growth factor-regulated tyrosine kinase substrate (HRS), which recognizes monoubiquitylated cargo proteins and associates in a complex with signal-transducing adaptor molecule (STAM), epidermal growth factor receptor pathway substrate 15 (Eps15), and clathrin. HRS recruits tumor susceptibility 101 (TSG101) of the ESCRT-I complex, and ESCRT-I is then involved in the recruitment of ESCRT-III through ESCRT-II or ALIX, an ESCRT-accessory protein. Finally, the dissociation and recycling of the ESCRT machinery require an interaction with the AAA-ATPase VPS4 [18,19].

The ESCRT-independent pathway includes ceramide-dependent manner, cluster of differentiation 63 (CD-63)-dependent mechanism, and ESCRT-independent exosomal nanovesicle formation in human embryonic kidney cells (HEK293) (Figure 1) [20]. For example, oligodendrocytes generate exosomes via the production of ceramide [16], while other cell types rely on the oligomerization of tetraspanin complexes [15,21].

Moreover, Rab GTPases, a family of conserved proteins that regulate vesicular trafficking and membrane fusion events, are also involved in exosome formation. Each member of the Rab GTPase family is differentially enriched in each subcompartment of an exosomal pathway: Rab5 is enriched in EVs; Rab7 and Rab 27 in MVBs; and Rab11, Rab25, Rab4, and Rab35 in the slow and rapid recycling routes. Rab9 is present in vesicles intended for the trans-Golgi network (TGN). Many viruses use these Rabs in various phases of the viral life cycle, although whether this usage influences exosomal release has not been investigated [11].

## 3. Exosome Composition

Exosomes are rich in a wide variety of proteins, lipids, and nucleic acids (Figure 1). Their unique composition depends on the type of cell and physiological or pathological state. It was reported that more than 50,000 proteins were found in exosomes, more than 164,000 messenger RNAs (mRNAs), and 12,000 microRNAs (miRNAs) in various types of membrane vesicles [22]. Analysis of exosomes from a wide variety of cells and body fluids has identified the following classes of proteins: (1) antigen presentation: Human leukocyte (HLA); (2) cell adhesion: integrins and Claudin-1; (3) cell structure and motility: actins, Cofilin-1, and Myosin; (4) heat shock proteins and chaperones: heat shock protein family A (Hsp70) member 8 (HSPA8); (5) metabolic enzymes: glyceraldehyde-3-phosphate dehydrogenase (GAPDH), phosphoglycerate kinase 1 (PGK1), and pyruvate kinase M2 (PKM2); (6) MVB biogenesis: ALIX, TSG101, vacuolar protein-sorting-associated proteins (VPS), and charged multivesicular body protein (CHMP); (7) signaling proteins: HRas proto-oncogene, GTPase (HRAS); (8) tetraspanins: CD9, CD63, CD81, and CD82; (9) transcription and protein synthesis: histones and ubiquitin; and (10) trafficking and membrane fusion: annexins, Ras-related protein Rab, ADP-ribosylation factor, and Synaptosomal-associated protein 23 [12]. In addition to a common set of proteins, as described above, cell-type-specific proteins that reflect the specialized function of the source cells seem to determine exosome functionality. For example, HIV-infected monocyte-derived macrophages release exosomes that contain the gag polyprotein [23].

Extracellular RNA can exist in two forms: in EVs or a nonvesicular form. The typical size of RNAs detected in EVs is 700 nucleotides (nt) [24], while miRNA contains 21 nt. The presence of functional RNAs in exosomes has been known for decades, and these RNAs can be delivered to and regulate gene expression in recipient cells [25,26]. The RNA content of exosomes seems similarly complex to their protein composition. Apart from larger mRNAs, mitochondrial RNA (mtRNA), long noncoding RNA (lncRNA), and lncRNA fragments, many small RNAs have also been detected: miRNA, miRNA fragments, small nuclear RNA (snRNA), and small nucleolar RNA (snoRNA, yRNA, vault RNA, fragments of transfer RNA (tRNA) and ribosomal RNA (rRNA)) [27]. The loading of miRNAs into exosomes is controlled by the heterogeneous nuclear ribonucleoprotein (hnRNP) A2B1, a ubiquitous protein involved in RNA trafficking and function. hnRNPA2B1 recognizes the EXO motif (GGAG tetranucleotide) in miRNAs and controls the loading of miRNAs containing this motif into exosomes [28].

Additionally, exosomal lipid composition has also been characterized, and exosomes are rich in sphingomyelin, gangliosides, phosphatidylserine, glycosphingolipids, and cholesterol [29]. These lipids may contribute to the stability of exosome formation. Additionally, some bioactive lipids can be transferred to and trigger downstream effects in recipient cells [29].

## 4. Roles of Exosomes in the Transmission and Replication of RNA Viruses

The association between exosomes and viruses is unclear. Current knowledge on the roles of exosomes in viral infection can be divided into two categories: facilitation and inhibition (Table 1). The former improves viral infectivity by spreading viral and cellular components, inducing immune evasion, and depressing the immune response [8]. Accumulating evidence indicates that host exosome pathways are hijacked by viruses, and virally modified exosomes contribute to the infection and immune evasion. The exosome biogenesis pathway has significant overlap with the assembly and egress of abundant viruses. In addition, many viruses utilize the exosomal pathway and interact with ESCRT proteins to augment replication processes, including assembly and egress [9].

### 4.1. Roles for Exosomes in Enveloped Virus Processes

Exosomes released from virus-infected cells are associated with a viral infection, host immunity, and microenvironmental modulation [11]. Exosomes secreted by enveloped viruses, such as HIV, hepatitis C virus (HCV), and human lymphotropic virus (HTLV), are attracting increasing attention [33,85,86].

#### 4.1.1. Exosomes with HIV

HIV is similar to exosomes in size, composition, and biogenesis. The density of exosomes ranges from 1.13 to 1.21 g·mL^−1^, while HIV-1 density ranges from 1.16 to 1.18 g·mL^−1^ [87]. In terms of size, HIV-1 is slightly larger than an exosome, with the diameter of the virus ranging from 100–120 nm compared to the 30- to 120-nm diameter of exosomes [87]. In addition, it is thought that HIV-1 can be formed by the exosome biogenesis pathway. Gould et al. even proposed a “Trojan exosome hypothesis,” which states that retroviruses, including HIV in particular, utilize the host exosome biogenesis and uptake pathway for the generation of infectious particles and a receptor- and Env-independent mode of infection [88]. However, evidence suggests that the relationship between exosomes and HIV is much more complicated. The role of exosomes in HIV infection depends not only on the infectious states of the original cell and the recipient cell but also on whether the exosome is derived from fluid or cells.

Several studies have documented the effects of exosomes in the context of HIV infection. Exosomes can carry a wide range of viral and host molecules including proteins, RNAs, chemokines, and cytokines to establish infection, complete assembly, support budding or escape the immune response. Chronic immune activation (CIA) and CD4^+^ T-cell depletion are two important markers of HIV-1 infection. There is substantial evidence that exosomes derived from HIV-1-infected cells participate in both processes [30]. Exosomes from HIV-1-infected macrophages assist in viral transfer to uninfected cells [31], and exosomes derived from infected dendritic cells (DCs) augment CD4^+^ T-cell infection [32]. Virus-modified exosomes can inhibit innate and acquired immunity. For example, exosomes from HIV-infected cells can silence the immune response to benefit viral replication through CD45, CD86, and major histocompatibility complex (MHC) II [33].

The proteins incorporated into exosomes also have critical roles in exosome actions. Exosomes that contain the HIV Nef protein, a multifunctional virulence factor, have various pathogenic effects, such as the induction of bystander CD4^+^ T-cell apoptosis [34], dysregulation of miRNAs that target genes in inflammation and HIV pathogenesis [35], and downregulation of the expression of cell-surface molecules such as MHC-I and CD4 for immune evasion [36]. The Nef protein also induces exosome secretion [35], thereby contributing to HIV/AIDS pathogenesis. Another viral protein often found in exosomes is HIV Gag [25].

Additionally, viral mRNA/miRNA and pathogen-associated RNAs, such as HIV transactivation response (TAR) RNA transcripts, found in exosomes can enhance viral infection and replication in recipient cells [37,38]. HIV infection of macrophages causes increased release of the viral miRNAs vmiR88, vmiR99, and vmiR-TAR in cell extracts and exosome preparations in vitro and in exosomes from the serum of HIV patients. In addition, vmiR88 and vmiR999 can induce TNF-α release via Toll-like receptor (TLR) 8 signaling in macrophages, which may induce chronic inflammation in HIV-positive patients [39].

Exosomes derived from HIV are rich in cytokines, chemokines, and chemokine receptors, which may explain the persistent immune activation observed. Exposure of naive peripheral blood mononuclear cells to exosomes purified from HIV-positive patients induces CD38 expression on naive and central memory CD4^+^ and CD8^+^ T cells, likely contributing to inflammation and viral proliferation via passive cell activation [40]. Exosomes secreted from HIV-infected cells carry the chemokine receptors CCR5 and CXCR, which can facilitate HIV budding and transmission [41]. Various host surface molecules (CD45, CD86, and MHC-II) are also transferred from HIV-infected cells via exosomes to constrain the immune response [42]. Primary human monocyte-derived exosomes enter endothelial cells and cause dysfunction via the TLR4 and NF-kB pathways, which induce the mRNA and protein expression of the adhesion molecule intercellular adhesion molecule 1 (ICAM-1), chemokine ligand (CCL)-2, and cytokine interleukin (IL)-6, which may contribute to heart disease in HIV infection and other diseases involving CIA [43]. More recently, Nef-induced exosome-associated ADAM17 was found to cause quiescent CD4^+^ T cells to become permissive to HIV-1 infection, whereas ADAM17 along with TNF-α could activate latent HIV-1 in primary CD4^+^ T lymphocytes and macrophages [89]. Some viruses utilize exosomes and recruit ESCRT members via interactions with short motifs, the late assembly domains that are located in ALIX and TSG101, and are related to virus budding and release [33]. Many viruses have been shown to use ESCRT for egress. HIV Gag interacts with tetraspanins, especially CD63 and CD81, to facilitate virion egress [87]. Exosomes derived from HIV-1-containing immature DCs can transfer HIV-1 to T cells without de novo infection [31]. However, the role of exosomes in HIV infection has not been fully elucidated, as exosomes can both facilitate infection and inhibit it [33].

Contrary to the evidence that the HIV-1-mediated exosomal pathway enhances infection and host defense evasion, there is evidence in support of a role for exosomes in inhibiting viral infections. Exosomes released from T cells have inhibitory effects on HIV. When compared to CD4-depleted exosomes derived from CD4^+^ T cells, CD4^+^ exosomes inhibit HIV-1 infection, which may be due to the disguising of HIV-1 envelope proteins by exosomal CD4, resulting in the inhibition of HIV infection. Similarly, exosomes secreted from CD8^+^ T cells can suppress HIV transcription within infected cells [44].

Exosomes can collect viral antigens and present these antigens to downstream recipient cells, inducing an antiviral immune response in the host [45]. Furthermore, the immunoregulatory factors released by immune cells, such as interferon (IFN)-α [46], IFN-β [47], TNF-α [48], and ILs [49], can be transferred by exosomes to the target site. For example, exosomes from TLR3-activated human brain microvascular endothelial cells (HBMECs) mediate the intercellular transfer of antiviral factors that inhibit HIV replication, including several key IFN-stimulated genes (ISGs: ISG15, ISG56, and Mx2), to macrophages at both the mRNA and protein levels [49]. APOBEC3G, a host cellular protein, transfers from cell to cell through exosomes to protect the recipient cell from HIV infection by restricting HIV replication through both DNA editing-dependent and editing-independent activities [50].

#### 4.1.2. Exosomes with HCV

Exosomes are strongly associated with HCV replication and infection. HCV occurs in both exosome-free and exosome-associated forms [51]. Exosome-associated HCV is infectious and resistant to neutralization by anti-HCV neutralizing antibodies [51]. However, exosomes in HCV have dual functions, with stimulatory effects on virus assembly, budding, spreading, and immune evasion and inhibitory effects related to antiviral immune defense.

Endosomal sorting complexes are responsible for transporting ESCRT-0 components, including HRS, and are involved in HCV budding [52]. ESCRT proteins chromatin-modifying protein 4B (CHMP4B) and tumor susceptibility gene 101 (TSG101), as well as the membrane trafficking regulator Annexin A2 (ANXA2), are required for HCV exosome release [52]. CD81 is an exosomal marker and the receptor for HCV, and it modulates the HCV envelope protein to facilitate release [53]. The endosomal pathway facilitates the sorting of HCV particles for release or degradation, and fully assembled HCV particles are either MVB-dependently released or degraded through the lysosome [12]. Complete HCV assembly machinery is typically required for HCV intercellular transmission; however, exosomes also have an assembly-free pathway to transfer HCV RNA for replication in uninfected cells [54].

HCV-modified exosomes facilitate infection in many ways. Exosomes derived from the serum of patients with chronic HCV infection or supernatants from HCV-infected cells contain replication-competent HCV RNA in complex with Ago2-miR122-HSP90, mediating viral receptor-independent transmission of HCV to recipient cells and the establishment of viral RNA replication in susceptible cells [55]. Interestingly, the GTPase Rab27a, which is related to the exosomal pathway, supports the HCV RNA replication process through a miR-122-mediated effect [56]. Moreover, HCV NS5A is associated with the core of low-density viral particles, which are released from cells through the exosomal pathway and may contribute to HCV infection [57]. Similarly, exosomes can protect HCV RNA from antibody neutralization, thus promoting infection [58].

However, contrary to their immune-evasive roles, exosomes containing HCV RNA can also stimulate innate immune responses in DCs [59]. HCV RNA alone cannot activate DCs; only when DCs internalize HCV exosomes via the endocytosis pathway or contact infected hepatocytes can IFN production be triggered in a manner dependent on viral RNA recognition by TLR7 [59]. HCV infection characterized by the induction of type I and type III IFNs and an abundance of DCs may mediate sustained inflammation in the liver [60]. HCV induces self-amplifying IFN-mediated responses and the release of exosomes with antiviral activity in human liver endothelial cells (HLSECs) [59]. Other anti-HCV effects involve the regulation of miRNAs. TLR3-activated macrophages confer anti-HCV activity to hepatocytes through exosomes, which were shown to contain anti-HCV miRNA-29 family members [61]. Moreover, exosomes secreted by umbilical mesenchymal stem cells inhibit HCV infection through the transfer of a mixture of miRNAs, including miR-145, miR-199a, and miR-221 released from exosomes [62].

#### 4.1.3. Exosomes and Other Enveloped Viruses

HTLV-1 is the causative agent of HTLV-1-associated myelopathy/tropical spastic paraparesis (HAM/TSP) and adult T-cell leukemia/lymphoma [63,64,65]. The transactivator protein Tax expressed by HTLV-1 affects many critical cellular pathways, including host cell DNA damage response mechanisms, cell cycle progression, apoptosis, and proinflammatory cytokines [63,64]. Recent results showed that the exosomes derived from HTLV contain functional HTLV-1 proteins (such as Tax protein) and mRNAs (tax, HBZ, and Env mRNAs), which can be transferred to recipient cells by exosome and stimulate the production of proinflammatory cytokines, such as IFN-γ, IL-6, and TNF-α, and T helper (Th)_1_-immune response [63,64,66,67]. Furthermore, exosomes produced by DCs or macrophages contain MHC class I and II molecules, as well as T-cell co-stimulatory molecules, which act as important factors in antigen presentation during HTLV-1 infection [9]. MHC I molecules incorporated into exosomes are responsible for the presentation of viral antigens, which contributes to the overall pro-inflammatory response associated with symptomatic HTLV-1 infection [66]. These results indicate that exosomes play critical roles during HTLV-1 infection to deliver host and viral components, and activate the immune response.

Moreover, multiple roles of exosomes were observed in other enveloped viruses. For example, exosomes from dengue virus (DENV)-2-infected cells contain IFN inducible transmembrane proteins 1, 2, and 3 (IFITM1, IFITM2, and IFITM3), which display antiviral activities in DENV-2-infected cells [68]. Human herpesvirus (HHV)-6 virions were encapsulated by MVBs, followed by egressing through the exosomal release pathway [90]. Influenza A virus, respiratory syncytial virus (RSV), and bunyaviruses attach to Rab11a-positive exosomes to reach the plasma membrane for egress [69].

## 5. Roles of Exosomes in Nonenveloped RNA Virus Processes

Apart from enveloped viruses, nonenveloped viruses, such as Hepatitis A virus (HAV), Hepatitis E virus (HEV), and enterovirus 71 (EV71), also need exosomes to participate in the infection.

HAV is a unique member of the *Picornaviridae* family that causes enterically transmission, and moderate to severe acute inflammatory liver injury [91]. Feng et al. identified exosome-like viral particles, which are characterized by incorporating HAV virions into exosomes with a diameter of 50–110 nm, and termed as quasi-enveloped HAV (eHAV) [70,71,72]. It was reported that phosphatidylserine receptor HAVCR1 (CD365) and cholesterol transporter NPC1 involve in the transportation of eHAV through clathrin-mediated endocytosis, which is necessary for the membrane fusion and transport of viral RNA from eHAV into the cytoplasm [73,74]. Moreover, host gangliosides are essential endosomal receptors for uncoating and delivery of the viral RNA genome to the cytoplasm of eHAV and naked HAV [72]. Further studies showed that HAV is mainly released in the form of eHAV by hijacking host ESCRT complexes via interaction between the viral structural protein pX and ALIX [70,71]. The eHAV are fully infectious and can be circulated in the blood of infected people, which is the only form of HAV that can be detected in the blood [70,72,75].

Similarly, HEV particles are individually covered by lipid membrane, which is similar to the exosomal membrane and enveloped viruses, and are released from infected cells through multivesicular body sorting and the cellular exosomal pathway [76,77,78,79]. Furthermore, exosomes from HEV-infected cells contain encapsidated HEV RNA, miRNA, cholesterol, phosphatidylserine, sphingomyelin, and ceramides, which are critical for the virus entry [80,81]. Moreover, exosomes also protect HEV particles from immune response, which leads to the widespread circulation of the virus in its host [80,81].

Moreover, it was reported that exosomes derived from EV71-infected rhabdomyosarcoma cells contain EV71 RNA, the viral capsid protein VP1 as well EV71 particles, which can establish a productive infection in vitro and are partially resistant to antibody neutralization [82,83]. However, exosomes from EV71-infected human oral epithelial cells preferentially package a high level of miR-30a, which can be delivered to the macrophages and inhibit type I IFN response via targeting myeloid differentiation factor 88 (MyD88), resulting in an enhancement of the virus replication [84].

## 6. Conclusions and Perspectives

Exosome functions partly depend on the cellular origin, but the target cells to which exosomes are delivered also play a pivotal role in viral pathogenesis. Therefore, the exact components of exosomes and their potential antiviral mechanism need further study.

Furthermore, mounting evidence has demonstrated that exosomes are critically relevant to specific diseases and treatment responses [92,93]. For instance, the level of serum exosomal CD81 was elevated in patients with chronic hepatitis C and associated with the severity of inflammation and fibrosis [93]. Additionally, the components in exosomes are relatively specific and stable [92]. Exosomes can be easily isolated from readily available biofluids, such as serum, blood, and urine [92]. Therefore, these results indicate that exosomes are promising candidates that can be used as diagnostic biomarkers and prognostic markers for virus infections and virus-related disease.

Moreover, exosome is an ideal vehicle for therapeutic applications by transferring interfering RNA, miRNA, and therapeutic compounds [94]. For example, shRNAs against a viral entry receptor and HCV replication machinery were transfected into several cell types, and the resulting shRNA-loaded exosomes mediated a significant decrease in HCV infection in liver cells [95]. In addition, miR-214 can be transported via exosomes to hepatocytes, resulting in decreased expression of CCN2, a gene known to be important in regulating liver fibrosis [95]. Besides, the exosomal pathway can also be targeted by novel inhibitors. As reported, exosomal inhibitors reduce the overall level of Env-dependent HIV infection [96]. Potential exosomal targets are cellular enzymes (RNases, proteases, and lipases), cytoplasmic or transmembrane proteins (TSG101, cyclophilins, MHC-II, and tetraspanins), and HIV-related proteins (e.g., Nef, CCR5, and CXCR4) [96].

## Figures and Tables

**Table 1:** The roles of exosomes in RNA virus biology.

Viruses	Possible Biological Roles	Reference
HIV	Helps to establish infection, complete assembly, support budding, and escape immune response	[30,31,32,33,34,35,36,37,38,39,40,41,42,43,44,45,46,47,48,49,50]
HCV	Affects virus assembly, budding, spreading and immune evasion	[51,52,53,54,55,56,57,58,59,60,61,62]
HTLV	Delivers host and viral components, activate the immune response, stimulate proinflammatory cytokines	[63,64,65,66,67]
DENV	Antiviral activities	[68]
IAV and RSV	Participates in viral egress	[69]
HAV	Involves in membrane fusion, transportation of viral RNA from eHAV into the cytoplasm, virus packing and releasing	[70,71,72,73,74,75]
HEV	Participates in virus entry, releasing, and escape immune response	[76,77,78,79,80,81]
EV71	Involves in virus infection, resistant to antibody neutralization, deliver miRNA	[82,83,84]
